# High-Throughput parallel blind Virtual Screening using BINDSURF

**DOI:** 10.1186/1471-2105-13-S14-S13

**Published:** 2012-09-07

**Authors:** Irene Sánchez-Linares, Horacio Pérez-Sánchez, José M Cecilia, José M García

**Affiliations:** 1 Computer Engineering Department, School of Computer Science, University of Murcia, Spain

## Abstract

**Background:**

Virtual Screening (VS) methods can considerably aid clinical research, predicting how ligands interact with drug targets. Most VS methods suppose a unique binding site for the target, usually derived from the interpretation of the protein crystal structure. However, it has been demonstrated that in many cases, diverse ligands interact with unrelated parts of the target and many VS methods do not take into account this relevant fact.

**Results:**

We present *BINDSURF*, a novel VS methodology that scans the whole protein surface in order to find new hotspots, where ligands might potentially interact with, and which is implemented in last generation massively parallel GPU hardware, allowing fast processing of large ligand databases.

**Conclusions:**

*BINDSURF *is an efficient and fast blind methodology for the determination of protein binding sites depending on the ligand, that uses the massively parallel architecture of GPUs for fast pre-screening of large ligand databases. Its results can also guide posterior application of more detailed VS methods in concrete binding sites of proteins, and its utilization can aid in drug discovery, design, repurposing and therefore help considerably in clinical research.

## Background

In clinical research, it is crucial to determine the safety and effectiveness of current drugs and to accelerate findings in basic research (discovery of new leads and active compounds) into meaningful health outcomes. Both objectives need to process the large data set of protein structures available in biological databases such as PDB [1] and also derived from genomic data using techniques as homology modeling [2]. Screenings in lab and compound optimization are expensive and slow methods, but bioinformatics can vastly help clinical research for the mentioned purposes by providing prediction of the toxicity of drugs and activity in non-tested targets, and by evolving discovered active compounds into drugs for the clinical trials.

This can be achieved thanks to the availability of bioinformatics tools and Virtual Screening (VS) methods that allow to test all required hypothesis before clinical trials. Nevertheless current Virtual Screening (VS) methods, like docking, fail to make good toxicity and activity predictions since they are constrained by the access to computational resources; even the nowadays fastest VS methods cannot process large biological databases in a reasonable time-frame. Therefore, these constraints imposes serious limitations in many areas of translational research.

The use of last generation massively parallel hardware architectures like *Graphics Processing Units *(GPUs) can tremendously overcome this problem. The GPU has become increasingly popular in the high performance computing arena, by combining impressive computational power with the demanding requirements of real-time graphics and the lucrative mass-market of the gaming industry [3]. Scientists have exploited this power in arguably every computational domain, and the GPU has emerged as a key resource in applications where parallelism is the common denominator [4]. To maintain this momentum, new hardware features have been progressively added by NVIDIA to their range of GPUs, with the *Fermi *architecture [5] being the most recent milestone in this path.

Therefore, GPUs are well suited to overcome the lack of computational resources in VS methods, accelerating the required calculations and allowing the introduction of improvements in the biophysical models not affordable in the past [6]. We have previously worked in this direction, showing how VS methods can benefit from the use of GPUs [7-9].

Moreover, another important lack of VS methods is that they usually take the assumption that the binding site derived from a single crystal structure will be the same for different ligands, while it has been shown that this does not always happen [10], and thus it is crucial to avoid this very basic supposition. In this work, we present a novel VS methodology called *BINDSURF *which takes advantage of massively parallel and high arithmetic intensity of GPUs to speed-up the required calculations in low cost and consumption desktop machines, providing new and useful information about targets and thus improving key toxicity and activity predictions. In *BINDSURF *a large ligand database is screened against the target protein over its whole surface simultaneously. Afterwards, information obtained about novel potential protein hotspots is used to perform more detailed calculations using any particular VS method, but just for a reduced and selected set of ligands. Other authors have also performed VS studies over whole protein surfaces [11] using different approaches and screening small ligand databases, but as far as we know, none of them have been implemented on GPUs and used in the same fashion as *BINDSURF*.

### Protein surface screening

The main idea underlying our VS method *BINDSURF *is the protein surface screening method, implemented in parallel on GPUs. Essentially, VS methods screen a large database of molecules in order to find which one fit some established criteria [12]. In the case of the discovery of new leads, compound optimization, toxicity evaluation and additional stages of the drug discovery process, we screen a large compound database to find a small molecule which interacts in a desired way with one or many different receptors. Among the many available VS methods for this purpose we decided to use protein-ligand docking [13,14]. These methods try to obtain rapid and accurate predictions of the 3D conformation a ligand adopts when it interacts with a given protein target, and also the strength of this union, in terms of its scoring function value. Docking simulations are typically carried out in a very concrete part of the protein surface in methods like Autodock [15], Glide [16] and DOCK [17], to name a few. This region is commonly derived from the position of a particular ligand in the crystal structure, or from the crystal structure of the protein without any ligand. The former can be performed when the protein is co-crystallized with the ligand, but it might happen that no crystal structure of this ligand-protein pair is at disposal. Nevertheless, the main problem is to take the assumption, once the binding site is specified, that many different ligands will interact with the protein in the same region, discarding completely the other areas of the protein.

Given this problem we propose to overcome it by dividing the whole protein surface into defined regions. Next, docking simulations for each ligand are performed simultaneously in all the specified protein spots. Following this approach, new hotspots might be found after the examination of the distribution of scoring function values over the entire protein surface. This information could lead to the discovery of novel binding sites. If we compare this approach with a typical docking simulation performed only in a region of the surface, the main drawback of this approach lies on its increased computational cost. We decided to pursue in this direction and show how this limitation can be overcome thanks to GPU hardware and new algorithmic designs.

In essence, in a docking simulation we calculate the ligand-protein interaction energy for a given starting configuration of the system, which is represented by a scoring function [18]. In *BINDSURF *the scoring function calculates electrostatic (ES), Van der Waals (VDW) and hydrogen bond (HBOND) terms. Furthermore, in docking methods it is normally assumed [12] that the minima of the scoring function, among all ligand-protein conformations, will accurately represent the conformation the system adopts when the ligand binds to the protein. Thus, when the simulation starts, we try to minimize the value of the scoring function by continuously performing random or predefined perturbations of the system, calculating for each step the new value of the scoring function, and accepting it or not following different approaches like the Monte Carlo minimization method [19] or others.

One of the main computational bottlenecks of docking simulation methods resides in the calculation of the scoring function [6]. We have already implemented non-bonded interactions kernels on GPUs [7] when direct summation is used to calculate the electrostatic interactions term, achieving speedups of up to 260 times versus the sequential counterpart. We will refer later to this kernel as *DIRECT_KERNEL*. When dealing with rigid or mixed flexible-rigid systems we can further improve the speed of the calculations using precomputed grids [20]. This kernel will be referred later as *GRID_KERNEL*. We studied the influence and convenience of both kernels for the design of *BINDSURF*, which was carried out totally from scratch on the GPU.

### Calculation of non-bonded interactions using grids

**Figure 1:**
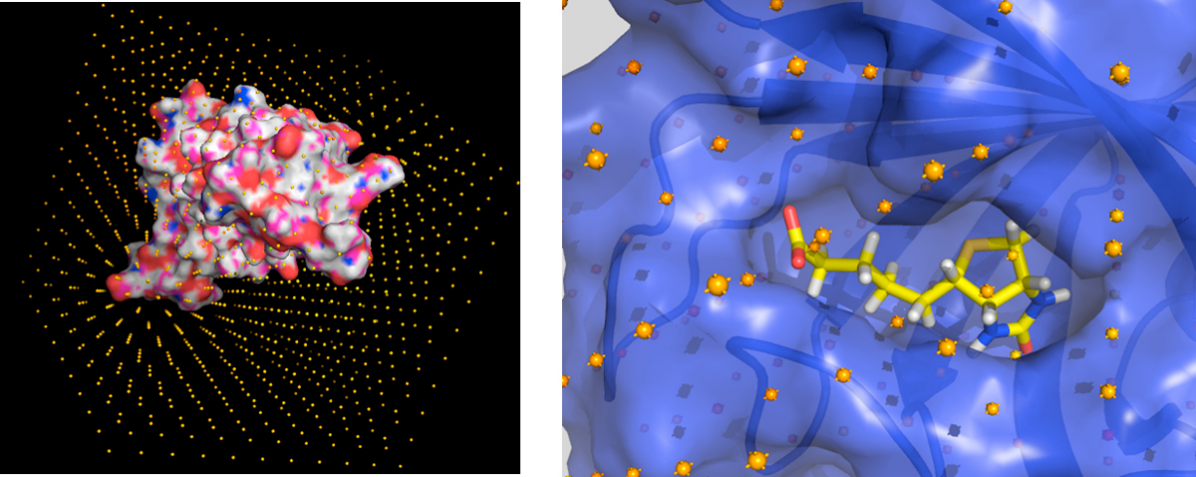
**Grid for streptavidin**. (A) Representation of the grid for the protein streptavidin. (B) Biotin in the crystallographic binding site of streptavidin.

*BINDSURF *uses ES, VDW and HBOND interaction kernels to calculate the scoring function of each ligand conformation for each step of the simulation during the whole energy minimization process. In case of the *GRID_KERNEL*, a grid approach is used for the calculation of the interactions. In the case of the ES part of the *GRID_KERNEL *kernel, we followed the initial idea described in [20] for the generation of protein grids and its latter application in the calculation of the non-bonded interactions. Both ES, VDW and HBOND grids are generated in CPU and GPU as described in [8]. Details about how the ES term is calculated on the GPU can be found in [9]. A graphical depiction of the grid for streptavidin is shown in Figure 1(A) and in more detail for the ligand biotin on its binding pocket in Figure 1(B).

### CUDA programming model

NVIDIA GPU platforms can be programmed using the Compute Unified Device Architecture (CUDA) programming model [21] which makes the GPU to operate as a highly parallel computing device. Each GPU device is a scalable processor array consisting of a set of SIMT (Single Instruction Multiple Threads) multiprocessors (SM), each of them containing several stream processors (SPs). Different memory spaces are available in each GPU on the system. The global memory (also called device or video memory) is the only space accessible by all multiprocessors. It is the largest and the slowest memory space and it is private to each GPU on the system. Moreover, each multiprocessor has its own private memory space called shared memory. The shared memory is smaller and also lower access latency than global memory.

The CUDA programming model is based on a hierarchy of abstraction layers: The *thread *is the basic execution unit that is mapped to a single SP. A *block *is a batch of threads which can cooperate together because they are assigned to the same multiprocessor, and therefore they share all the resources included in this multiprocessor, such as register file and shared memory. A *grid *is composed of several blocks which are 5 equally distributed and scheduled among all multiprocessors. Finally, threads included in a block are divided into batches of 32 threads called *warps*. The warp is the scheduled unit, so the threads of the same block are scheduled in a given multiprocessor warp by warp. The programmer declares the number of blocks, the number of threads per block and their distribution to arrange parallelism given the program constraints (i.e., data and control dependencies).

## Methods

**Algorithm 1 ***BINDSURF *overview

1: Read main simulation configuration file *bindsurf_conf.inp*

2: Generate ES and VDW grids (*es_grid*, *vdw*_*grid*) of the protein using *GEN_GRID*

3: Generate *ligand*_*conformations *with *GEN*_*CONF*

4: Read *protein *and calculate *surface*_*spots *using *GEN*_*SPOTS*

5: **for all ***ligand_conformations ***do**

6:    Calculate *initial_configuration *of the system on GPU (*protein*, *surface_spots*, *ligand_conformation*) using *GEN_INI*

7:    Surface Screening using *SURF_SCREEN *(*initial_configuration*, *ligand_conformation*, *protein*, *surface_spots*, *es_grid*, *vdw_grid*)

8: **end for**

9: Process results

This Section introduces the underlying design of *BINDSURF *which is summarized in Algorithm 1. All necessary simulation parameters are specified in a configuration file called *bindsurf_conf.inp*which contains the following information:

• **Molecular data: **Filenames related with protein, ligand and input-output directories. Also, number of ligand conformations that will be generated from the input one.

• **Force field data**: ES, VDW and HBOND force field parameters used in the scoring function. By default we provide the OPLS force field [22].

• **Monte Carlo minimization parameters**: number of steps of the simulation, energy cutoff, maximum values for random shift and rotation.

• **Output related data**: file names for the different output files such as for graphical display through Pymol [23], energy distributions, detailed information about the poses with the lowest scoring function values, etc.

Once *bindsurf_conf.inp*has been read and processed, we generate an ensemble of ligand conformations from the input ligand using *GEN_CONF*; an ad hoc version of the FlexScreen docking program [24]. Given the modular structure of *BINDSURF*, any other program which generates ligand conformational ensembles can be used for the same purpose.

Next, we need the information pertaining to the areas of the protein surface (spots) where each individual simulation will take place. It is important to find an optimal number of spots, since too many spots would increase unnecessarily the total computation time, and few spots would not cover completely the protein surface. We have found that a good strategy, implemented in *GEN*_*SPOTS*, consists in the calculation for the input protein of the coordinates of the alpha carbons of each residue. All parallel simulations will take place in spherical regions, defined by the centers of these spots, with a cutoff radius of 10 Angstroms. This module can be easily changed to any other that just outputs the list of the coordinates of the spots.

In the fourth step of Algorithm 1 we generate the electrostatic (ES) and van der Waals (VDW) grids of the protein using the GPU program *GEN_GRID*, as depicted in our previous work [8]. The main idea underlying *GEN_GRID *is to suppose that the protein is rigid, and then to precomputate the electrostatic potential and the neighbours lists, in a regular spatial disposition of points called grid [20], as depicted in, that comprises both the protein and the ligands. *GEN_GRID *reads its parameters from the configuration files, generates the grids belonging to the input protein and writes them into different output files that are consequently processed during conformation generation and surface screening, resulting in fast computations of the scoring function.

Before the surface screening process can begin, we need to generate all necessary simulation parameters from the input ligand and protein with the GPU program *GEN_INI*. For a given ligand *ligand_conformation*, *GEN_INI *performs random ligand translations and rotations in order to obtain valid starting conformations of the ligand on each protein surface spot. Once the system is set up, the program *SURF*_*SCREEN *performs the protein surface screening procedure. Finally, *BINDSURF *reports statistics of the obtained results, Pymol files for its convenient 3D visualization as well as many other reports, as will be shown in the results section.

### Surface screening on GPU

In this Section, we introduce the main core of the *BINDSURF *program (namely *SURF_SCREEN*). Algorithm 2 shows the host side pseudocode of *BINDSURF*. Firstly, the previously obtained information regarding protein and its precomputed grids and surface spots, the ligand conformation, and the simulations initial states are transferred from CPU to the GPU, where all the simulation process takes place.

**Algorithm 2 **Host side of the *SURF_SCREEN *core of the *BINDSURF *application for a given ligand conformation

1: *CopyDataCPUtoGPU *(*protein*, *es_grid*, *vdw_grid*, *surface_spots*, *ligand_conformation; shifts_set*, *quaternions_set*, *initial_configuration*)

2: **for ***i *= 0 to *number_of_spots/BLOCKSURF ***do**

3:    **for ***n *= 1 to *numSteps ***do**

4:       **if **n is even **then**

5:          *GenerateShiftsKernel *(*randomStates*, *shifts_set*)

6:       **else**

7:          *GenerateRotationsKernel*(*randomStates*, *quaternions_set*)

8:       **end if**

9:       *Energy*(*es_grid*, *vdw_grid*, *protein*, *ligand_conformation*, *shifts_set*, *quaternions_set*, *newEnergy*)

10:       **if **n is even **then**

11:          *UpdateShiftsKernel*(*randomStates*, *oldEnergy*, *newEnergy*)

12:       **else**

13:          *UpdateRotationsKernel*(*randomStates*, *oldEnergy*, *newEnergy*)

14:       **end if**

15:    **end for**

16:    *FindMinima*(*oldEnergy*, *minIndexes*, *minEnergy*)

17:    *CopyDataGPUtoCPU*(*minIndexes*, *minEnergy*, *shifts_set*, *quaternions_set*)

18: **end for**

On each spot, many simulations (in this case, 128) for each ligand conformation are carried out in parallel on the GPU. The protein-ligand interaction energy is minimized using a parallel adaptation of the Monte Carlo method, utilizing the Metropolis algorithm [19]. Required random numbers in Monte Carlo are generated using the NVIDIA CURAND library [21], which later are employed to perform the required ligand rotations and displacements in parallel.

As a minimization process, the next iteration always depends on the previous one. Thus, the loop comprised between steps 3 to 15 in the Algorithm 2 is not affordable for parallelization. Therefore, only the internal computation is paralellized; i.e. the generation of shifts and rotations, energy calculation and the update of simulation state.

Moreover, we cannot launch simultaneously all the threads we need for the execution of all the simulations in parallel because the number of threads needed is greater than the maximum allowed. Hence, we only perform simulations for a maximum number of spots (BLOCKSURF value) simultaneously (line 2 in the Algorithm 2).

On one hand, each simulation needs to have a copy of the ligand that can modify. On the other hand, the number of simulations required in this process is huge, and thus it is not viable to have a copy of all information related to the ligand atoms in the GPU memory, such as for instance, all the atom positions. An alternative way of representing the ligand information, which is independent of the ligand size and thus benefits its allocation in the scarce internals of the GPU memory, is to keep a model of the ligand in the GPU constant memory. In this way, the state of each simulation is represented by one 3D point and a quaternion which represent the displacement and rotation about the origin, accumulated along the simulation. This solution can be applied because we use a rigid representation of the molecules.

The Monte Carlo process alternates different steps of rotation and displacement. Thus, we developed two different kernels; (1) for generating displacements of the simulations (called *GenerateShiftsKernel*), and (2) for generating rotations (called *GenerateRotationsKernel*). These kernels generate a random move using a local copy of the random state of each simulation and do not modify the random state in global memory. Later, if that move is accepted, the random state in global memory is updated with the random state that generated this movement; otherwise, the random state is not updated and the move is undone in the simulation state.

The function *Energy *in Algorithm 2 launchs highly GPU optimized non-bonded interaction kernels [9] for the description of the electrostatic, Van der Waals and hydrogen bond interactions between the ligand and the protein. These kernels are named *ESKernel *and *VDWKernel*, which are described in posterior sections. Once the energy is calculated, *UpdateShiftsKernel *and *UpdateRotationsKernel *check whether the previous energy values are smaller than the new values calculated for the energy, and if so the movement made is applied permanently to the simulation state.

The minimum value found for the energy belonging to the same sphere surface is obtained by *FindMinima *function. This function launches two different kernels; (1) a kernel to reduce the energy vector, which stores all energy values calculated in all simulations, and (2) a kernel to compact this vector, in order to reduce the data transferred to the CPU. Finally, we obtain a vector which contains the minimum energy obtained in the simulations for each spot.

Once the simulations are carried out on each protein spot in the surface, in the final output *BINDSURF *produces for each ligand detailed information about the protein spots where the strongest interactions are found for the different ligand conformations. This information can be parsed directly to PyMOL [23] to get a graphical depiction of the results. The information regarding the hotspots obtained for different ligands, and the set of ligands with the lowest values of the scoring function, is thought to be later employed in a more detailed VS methodology to screen only this resulting set of ligands in the hotspots found by *BINDSURF*. Other option is to pass the resulting ligand binding pose obtained by a detailed VS method for a binding site as input for *BINDSURF *to check whether it could interact in other parts of the protein surface.

In the next subsections we describe how is the scoring function calculated in both CPU and GPU versions.

### ElectroStatic (ES) energy calculation

#### Sequential baselines

**Algorithm 3 **Sequential pseudocode for the calculation of the electrostatic potential

1: **for ***i *= 1 to *N*_*simulations ***do**

2:    **for ***j *= 1 to *nlig ***do**

3:       *energy*[i * *nlig *+ *j*] = q_i _* *interpolate *(*lig*[i * *nlig *+ *j*], *ESGrid*)

4:    **end for**

5: **end for**

The precomputed ES grid is generated by the program *GEN_GRID *as described in [8] and afterwards, it is read by *SURF_SCREEN *from file and loaded onto memory. The calculation of the electrostatic potential for the protein-ligand system is performed as follows; for each ligand atom *i *with charge *q_i _*at point *P_i _*we calculate the eight closest protein grid neighbours. Next, an interpolation procedure is applied to estimate the value of the electrostatic potential due to all protein atoms at *P_i_*. The same procedure is applied to all ligand atoms summing them up. The pseudocode is shown in Algorithm 3, where *N_simulations *is the number of simulations, *nlig *is the number of atoms of each ligand and the function *interpolate *performs the calculation of the electrostatic potential for each atom.

#### GPU design

**Algorithm 4 **GPU pseudocode for the calculation of the electrostatic potential

1: **for all **nBlocks **do**

2:    *dlig *= *rotate*(*clig*[*myAtom*], *myQuaternion*)

3:    *ilig *= *shift*(*myShift*, *dlig*)

4:    *index *= *positionToGridCoordinates *(*ESGridInfo*, *ilig*)

5:    *energy*_*shared*[*myAtom*] = *charge*[*myAtom*] * *accessToTextureMemory*(*ESGrid*, *index*)

6:    *totalEnergy *= *parallelReduction*(*energy_shared*)

7:    **if ***threadId *== *numThreads*%*nlig ***then**

8:       *energy*[*mySimulation*] = *totalEnergy*

9:  **end if**

10: **end for**

In a previous work [9], we studied different strategies for the GPU implementation of the previous algorithm applied to many different ligands. In that study, we reported that the use of the texture memory decreases considerably the time needed for the calculation of the interpolation. Therefore, in *BINDSURF *we decided to use the texture memory to obtain the electrostatic potential by linear interpolation in a 3D grid. The Algorithm 4 shows the pseudocode of the ES kernel, where *clig*, *charge *and *nlig *is the ligand model (atom positions and charges, and number of atoms); *myAtom*, *myQuaternion *and *myShift *are the atom assigned to the thread and the rotation and shift belonging to the simulation assigned to the thread *mySimulation*; *ESGridInfo *and *ESGrid *are the grid description and the grid data (the latter is stored in the GPU texture memory) and *energy_shared *is a auxiliary vector in shared memory.

Each thread calculates the energy of only one atom. Firstly, each thread has to obtain the current atom position from the ligand model and the simulation state using the functions *shift *and *rotation*. Then, it calculates the grid position (function *positionToGridCoordinates*), interpolates the energy value accessing to the texture memory and (function accessToTextureMemory) stores the result in shared memory (line 5). Finally, threads of the same simulation sum up their results by a parallel reduction (line 6) with complexity order *O*(*log*(*n*)) and one of these threads writes the final result in global memory (lines 7-8).

### Van der Waals (VDW) and Hydrogen Bonds (HBOND) energies calculation

#### Sequential baselines

**Algorithm 5 **Sequential pseudocode for the calculation of the VDW and HBOND energies

1: **for ***i *= 1 to *N_simulations ***do**

2:    **for ***j *= 1 to *nlig ***do**

3:       *index *= *positionToGridCoordinates*(*V DWGRidI n f o*, *j*)

4:          **for ***k *= 0 to *numNeighbours*(*V DWGRid*[*index*]) **do**

5:          *vdwTerm*+ = *vdwEnergy *(*j*, *V DWGRid *[*index*][*k*])

6:          *hbondTerm*+ = *hbondEnergy *(*j*, *V DWGrid*[*index*][*k*])

7:       **end for**

8:   **end for**

9:    energy[i * nlig + j] = vdwTerm + hbondTerm;

10: **end for**

The precomputed VDW grid is generated by the program *GEN_GRID *as described in [8] and afterwards, it is read by *SURF*_*SCREEN *from file and loaded onto memory. Next, the Van der Waals (VDW) energy of each atom is calculated using the expression explained before and following the pseudocode shown in Algorithm 5, where *N _simulations *is the number of simulations, *nlig *is the number of atoms of the ligand and the functions *vdwEnergy *and *hbondEnergy *performs the calculation of the Van der Waals and hydrogen bonds potentials for each pair of atoms.

#### GPU design

The Algorithm 6 shows the pseudocode of the VDW kernel, where *V DWGridInfo *and *V DWGrid *are the grid description and the grid data, both stored in the GPU global memory. Other variables have the same meaning than in Algorithm 4. Each thread calculates the energy of only one atom. In the same way as the previous kernel, each thread applies the rotation and displacement corresponding to the simulation over the ligand model in order to obtain the current atom position (lines 2-3). Then, it calculates the grid position, calculates the VDW and HBOND potentials using the neighbors stored in the VDW grid and stores the result in shared memory (lines 4-9) . The parameters needed by the VDW and HBOND energies are previously stored in the GPU constant memory. Finally, threads of the same simulation sum up their results by a parallel reduction (line 10) and one of these threads accumulates the final result in global memory (lines 11-12).

**Algorithm 6 **GPU pseudocode for the calculation of the VDW and HBOND energies

1: **for all **nBlocks **do**

2:    *rlig *= *rotate*(*clig*[*myAtom*], *myQuaternion*)

3:    *ilig *= *shift*(*myShift*, *rlig*)

4:    *index *= *positionToGridCoordinates*(*V DWGridI n f o*, *ilig*)

5:    **for ***k *= 0 to *numNeighbours*(*V DWGRid*[*index*]) **do**

6:       *vdwTerm*+ = *vdwEnergy*(*j*, *V DWGRid*[*index*][*k*])

7:       *hbondTerm*+ = *hbondEnergy*(*j*, *V DWGrid*[*index*][*k*])

8: **end for**

9: *energy*_*shared*[*myAtom*] = *vdwTerm *+ *hbondTerm*

10: *totalEnergy *= *parallelReduction*(*energy*_*shared*)

11: **if ***threadId *== *numThreads*%*nlig ***then**

12:       *energy*[*mySimulation*]+ = *totalEnergy*

13: **end if**

14: **end for**1

## Results and discussion

### Experimental setup

Particular features for our hardware and software equipment are summarized in Tables 1 and 2. GPUs are plugged into the motherboard using PCI-express 2 when required.

**Table 1:** Hardware features

	Processors for a $3000 high-end server	
	
	CPU	GPU
Release date	Q4 2009	Q4 2009
Codename	Intel Westmere	Nvidia Fermi
Estimated cost	$500	$1500

Commercial model	Xeon E5620	Tesla C2050
No. cores @ speed	4 @ 2.4 GHz	-
No. stream processors	-	448 @ 1.15 GHz

L2 cache size	12 MB.	768 KB. .
DRAM memory size	16 GB.	3 GB.
DRAM type	DDR3	GDDR5

Memory bus width	128 bits	384 bits
Memory clock	1066 MHz	2 × 1.5 GHz
Memory bandwidth	17 GB/s	144 GB/s

Summary of hardware features for the CPUs and GPUs used during our experimental survey.

**Table 2:** Software resources

Target hardware	Software tools
Intel Xeon CPU	gcc compiler, 4.3.4 version with the -O3 flag

Nvidia Tesla GPU	CUDA compilation tools, release 4.0

Software resources used for each hardware platform in our experimental study.

### Performance measures

**Figure 2:**
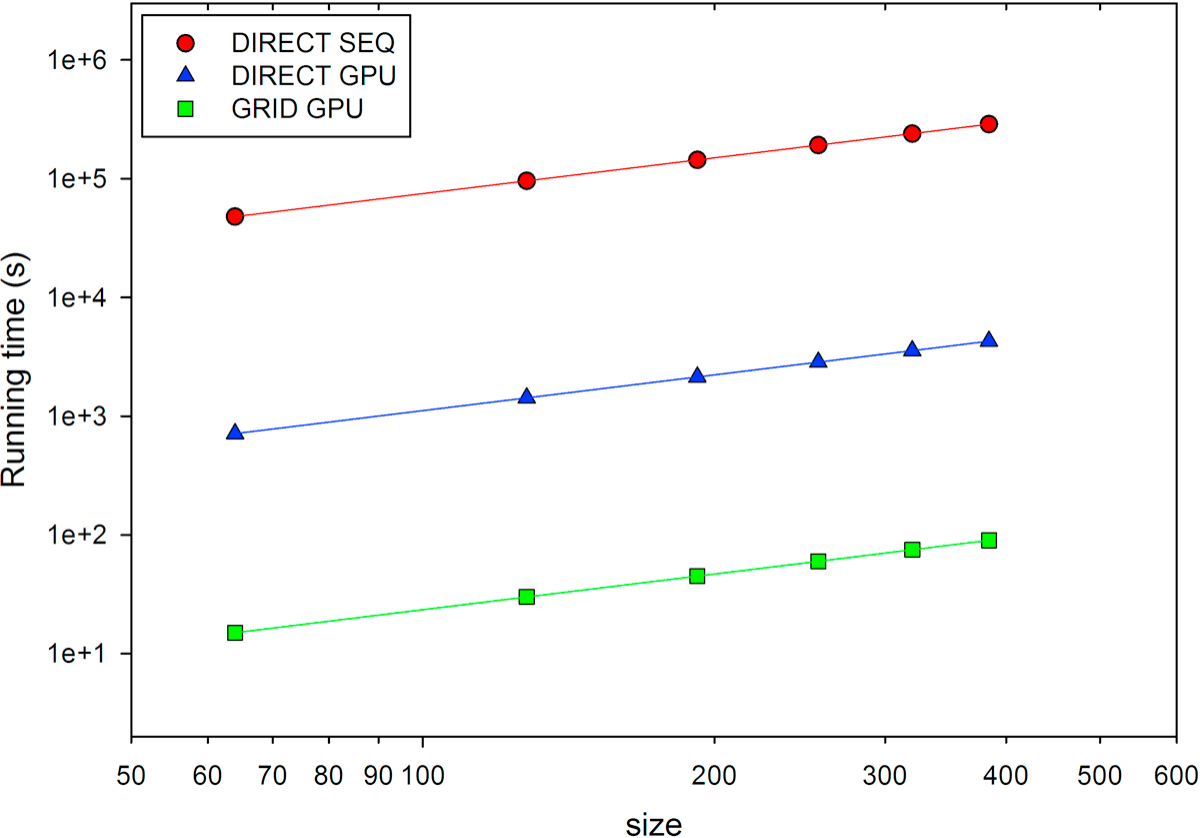
**Performance of *BINDSURF***. Running times for the different implementations of *BINDSURF*, when performing surface screening over the protein PDB:1M54 and increasing the number of processed spots, specified by the parameter *size*. SEQ and GPU denote sequential and GPU versions respectively. DIRECT and GRID refer to the respective kernels.

We measured the performance of *BINDSURF *in the form of total running time, for its different implementations depending on the kernel used; SEQ and GPU, which denote sequential and GPU versions respectively, and DIRECT or GRID, as explained in the Methods Section. When GRID is used, the optimal value for the separation between grid points is equal to *d *= 0.5 · Å, as reported previously [8]. Surface screening was performed over the protein PDB:1M54. Simulations were always carried out with a total of 500 Monte Carlo steps. Running times were obtained while increasing the number of processed spots, specified by the parameter *size*. Observing Figure 2 it is clear that for all implementations the total running time depends linearly on the size of the system. But it should be noticed that if reduced the value of the distance between grid points, *d*, the number of grid points would increase and the GRID GPU and GPU SEQ kernels would run slower. Additionally, the GPU version outperforms the SEQ version in the case of the DIRECT kernel, thanks to the very efficient DIRECT kernel, which was optimized specifically for GPU [7]. Finally, we can observe how the GRID GPU kernel outperforms also the DIRECT GPU kernel, being the fastest implementation of *BINDSURF*, and the one we used for all the application cases explained later.

The performance of *BINDSURF *was also determined through the measure of the partial running times for the different programs that form *BINDSURF *(*GEN*_*GRID*, *GEN*_*CONF*, *GEN*_*INI *and *SURF*_*SCREEN*). In Table 3 we can observe the results obtained for one single ligand conformation and one single receptor (PDB:1M54). As we increase the size of the system, the total running times of *GEN*_*GRID*, *GEN*_*CONF *and *GEN*_*INI *remain approximately constant, and on overall, start to contribute less to the total running time, while *SURF*_*SCREEN *becomes the most computationally intensive part of *BINDSURF *(*SURF*_*SCREEN*). The program *GEN*_*CONF *generates by default 100 different conformations for the input ligand. Since *GEN*_*CONF *is a non-optimized sequential program, it needs too much time (among 40 and 80 % of the total running time) for its processing and its use should be avoided. Given the modular design of *BINDSURF*, the user can substitute it for any other generator of ligand conformations, in the desired case of use of a conformational ensemble of ligand conformations to represent ligand flexibility. Nevertheless, at this stage *BINDSURF *is optimized for small or medium size ligands which can be modelled without consideration of the flexibility, and we leave its implementation for a next stage. We conclude therefore in this part that the ideal application scenarios of *BINDSURF *are the screening of single rigid ligand conformations over the whole surface of rigid receptor models (or ensembles of receptor conformations that might represent partially the flexibility of the protein), given its ultra fast processing speed, of around one minute for ligand-receptor pair. To the best of our knowledge, there is no other method that can perform protein surface screening in these conditions and at such speed. Other ideal application scenarios would be the case of multi target drug screening [8,25], useful for toxicity prediction, and fragment based (since they can efficiently be modeled without flexibility consideration) drug screening (in a similar fashion as depicted in [26]) over the whole protein surface.

**Table 3:** Running times for the different parts of BINDSURF

size	*% GEN_GRID*	*% GEN_CONF*	*% GEN_INI*	*% SURF_SCREEN*	*BINDSURF *running time
64	7.8	82.7	2.7	6.8	29.4

128	6.2	64.8	2.9	26.1	37.5

192	5.0	51.1	3.6	40.3	47.6

256	4.5	45.6	3.8	46.2	53.3

320	4.1	41.8	3.6	50.4	58.1

356	3.6	38.0	3.9	54.5	63.9

Percentage of the running times for the different parts of *BINDSURF *(*GEN*_*GRID*, *GEN*_*CONF*, *GEN*_*INI *and *SURF*_*SCREEN *) and total *BINDSURF *running time value (in seconds) when changing the size of the system (total number of spots, first column) processed for PDB:1M54.

### Applications

*BINDSURF *does not make any assumption about the location of the binding site of the protein. After a *BINDSURF *execution, and with the obtained information about how different ligands dock in the protein surface we can start to make hypothesis about different potential binding sites which can guide posterior and more detailed analysis using known standard docking tools like Autodock [15], FlexScreen [24], Glide [16] or DOCK [17], or Molecular Dynamics or mixed Quantum-Mechanical/Molecular-Mechanics methods. We run *BINDSURF *over receptor-ligand structures selected from the PDB database, which are known to have one or several binding pockets depending on the ligand, and checked whether *BINDSURF *could find correctly a) the binding site area, b) the binding pose of the crystallographic ligand. Simulations were always carried out with a total of 500 Monte Carlo steps. We also selected application cases known to present difficulties for binding site prediction with other methods.

**Figure 3:**
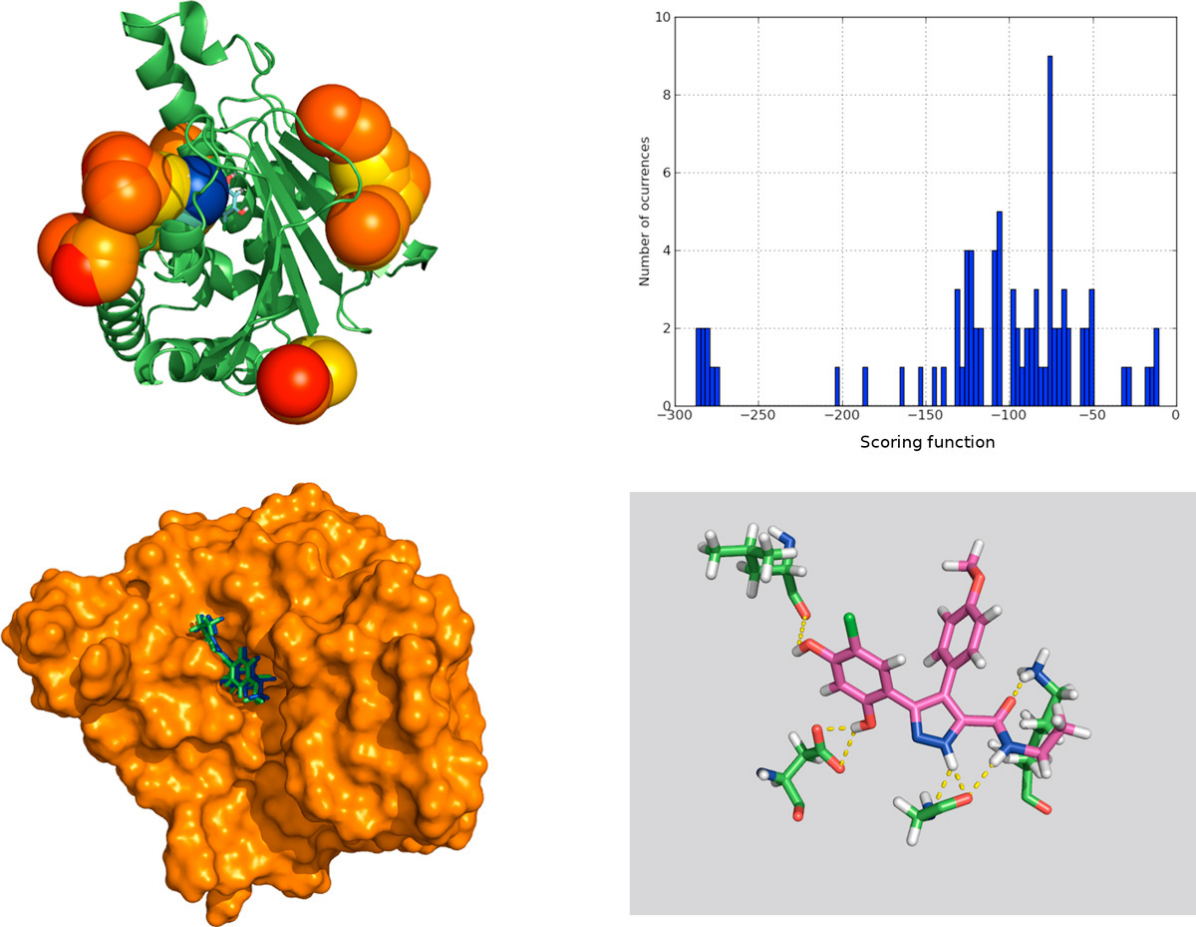
**Surface screening results for PDB:**2BSM. Surface screening results for PDB:2BSM. From up left to down right; a) beads represent protein spots and the color of each bead is related with the value of the scoring function, so colors from red to blue indicate lower values for the scoring function, b) histogram with the distribution of scoring function values, c) green and blue molecules represent crystallographic and predicted pose for the ligand, RMSD is lower than 1 Angstrom, and d) depiction of the hydrogen bonds established by the ligand with the closest residues.

We also obtained concordance with some other methods that try to predict the binding site based on the protein structure alone [27]. In Figure 3.a, we show how the strongest interaction spot (blue sphere) for chaperone Hsp90 (PDB:2BSM) coincides with crystal binding site. Figure 3.b shows how its scoring function value is clearly differentiated from the other weak interaction spots, with higher value for the scoring function. The shape of the binding pocket is shown in Figure 3.c, where we can observe that predicted and crystallographic binding poses coincide rather well, with RMSD lower than 1 Angstrom. Finally, Figure 3.d shows the hydrogen bond network predicted by *BINDSURF *for the ligand.

**Figure 4:**
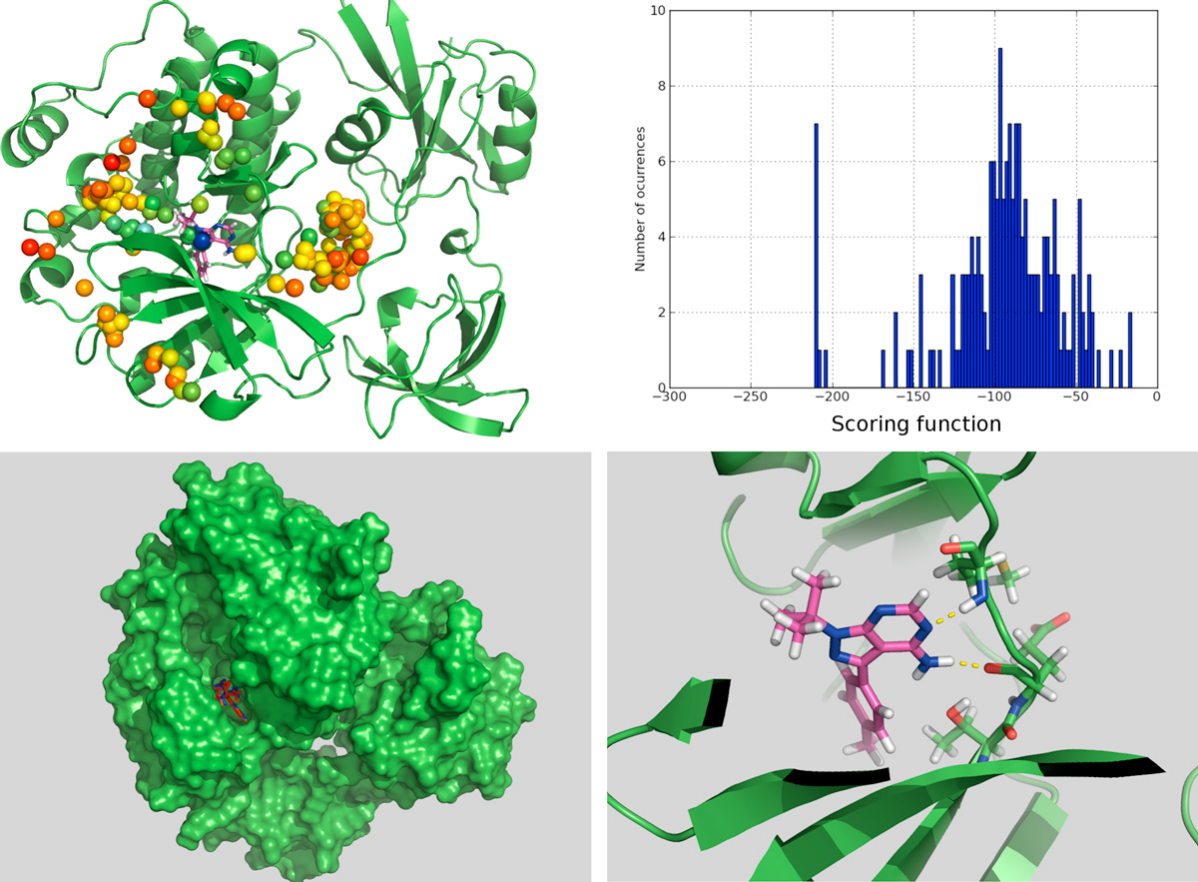
**Surface screening results for PDB:1QCF**. Surface screening results for PDB:1QCF. From up left to down right; a) beads represent protein spots and the color of each bead is related with the value of the scoring function, so colors from red to blue indicate lower values for the scoring function, b) histogram with the distribution of scoring function values, c) red and blue molecules represent crystallographic and predicted pose for the ligand, RMSD is lower than 1 Angstrom, and d) depiction of the hydrogen bonds established by the ligand with the closest residues.

We compared also with the final binding site found and ligand binding poses obtained using very long trajectories in Molecular Dynamics simulations in Supercomputers [28-30] for a tyrosine kinase protein (PDB:1QCF). It must be noticed that with *BINDSURF *the dynamical information about the binding process cannot be obtained. In Figure 4, we can observe the accuracy of the prediction of *BINDSURF *both for the location of the binding site, and for the prediction of the ligand pose.

**Figure 5:**
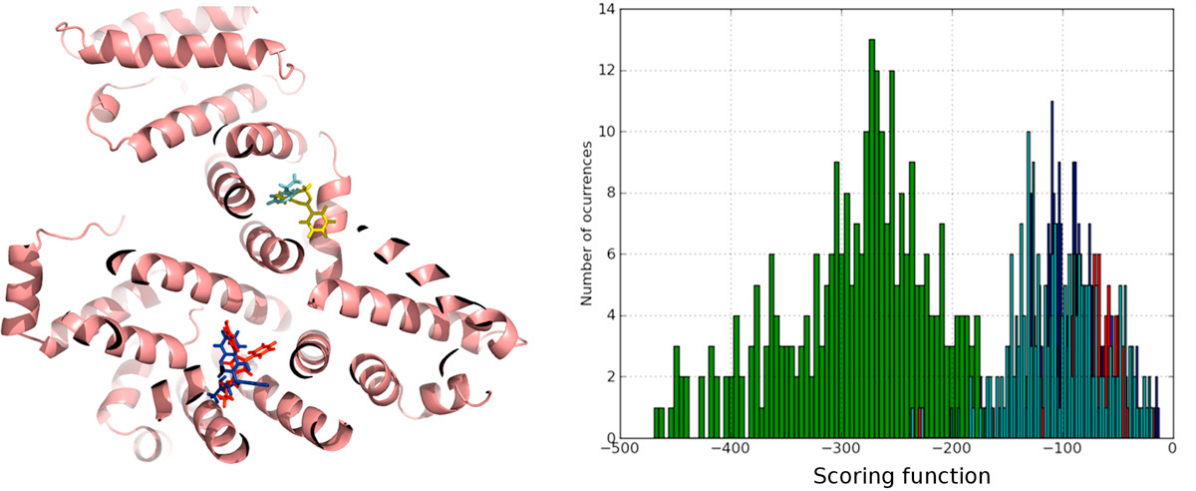
**Surface screening results for proteins PDB:**2BXB, PDB:2BXD, PDB:2BXF, PDB:2BXG. Surface screening results for proteins PDB:2BXB, PDB:2BXD, PDB:2BXF, PDB:2BXG. From left to right; a) ligand poses predicted by *BINDSURF *for the ligands of proteins PDB:2BXB (red color), PDB:2BXD (dark blue color), PDB:2BXF (yellow color) and PDB:2BXG (light blue color), with average RMSDs less than 2 Angstroms, and b) histogram with the distribution of scoring function values for PDB:2BXG (green color), PDB:2BXB (red color), PDB:2BXD (light blue color), and PDB:2BXF (dark blue color).

**Figure 6:**
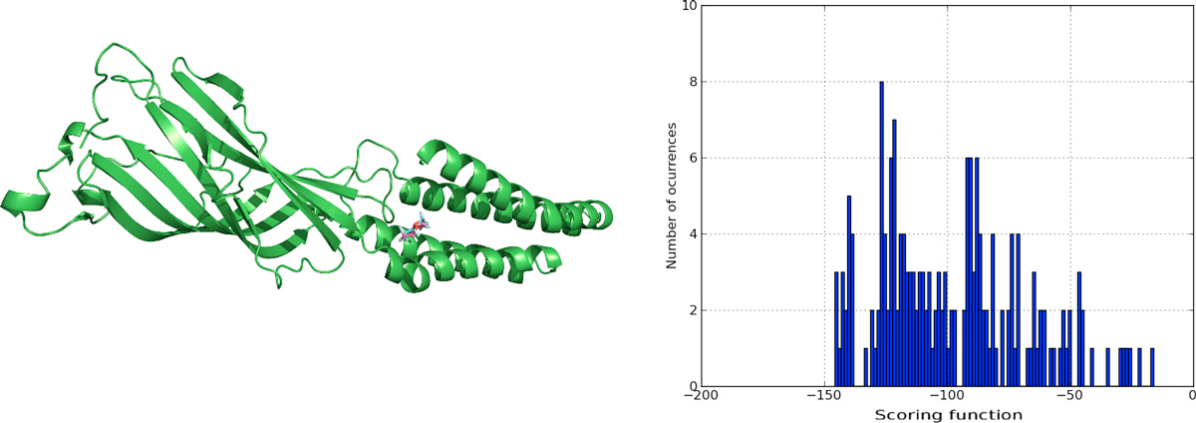
**Surface screening results for PDB:**3P4W. Surface screening results for PDB:3P4W. From left to right; a) superposition of predicted and crystallographic ligand poses, with RMSD less than 2 Angstroms, and b) histogram with the distribution of scoring function values.

**Figure 7:**
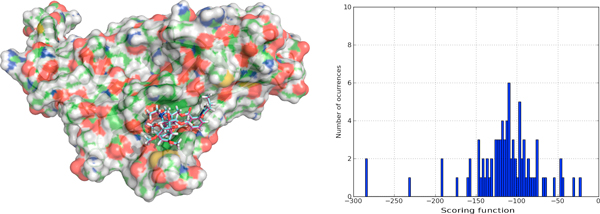
**Surface screening results for PDB:**2BYR. Surface screening results for PDB:2BYR. From left to right; a) superposition of predicted and crystallographic ligand poses, with RMSD less than 3 Angstroms, and b) histogram with the distribution of scoring function values.

There have been previous studies [31] in human serum albumin (HSA) where it has been shown the existence of two primary drug-binding sites on the protein, and other secondary binding sites for drugs distributed across the protein. In Figure 5.a we show that *BINDSURF *can predict the binding site and binding pose in HSA when it is complexed with different ligands (cases PDB:2BXB, PDB:2BXD, PDB:2BXF, PDB:2BXG). Figure 5.b shows that it obtains different scoring function distribution profiles depending on the ligand. Thus, our method can work efficiently also for multiple binding site prediction. We decided also to test predictions for ion channel proteins, where some docking methods fail given the narrow internal cavity of the protein. In Figure 6 the prediction results for a pentameric ligand-gated ion channel, GLIC, (PDB:3P4W) are shown, and manifest good agreement with the experimental result. In the case of acetylcholine-binding proteins (PDB:2BYR), which provide very useful information for the modeling of the extracellular domain of pentameric ligand-Gated ion channels, we also obtained good agreement with the experimental results, as shown in Figure 7.

**Figure 8:**
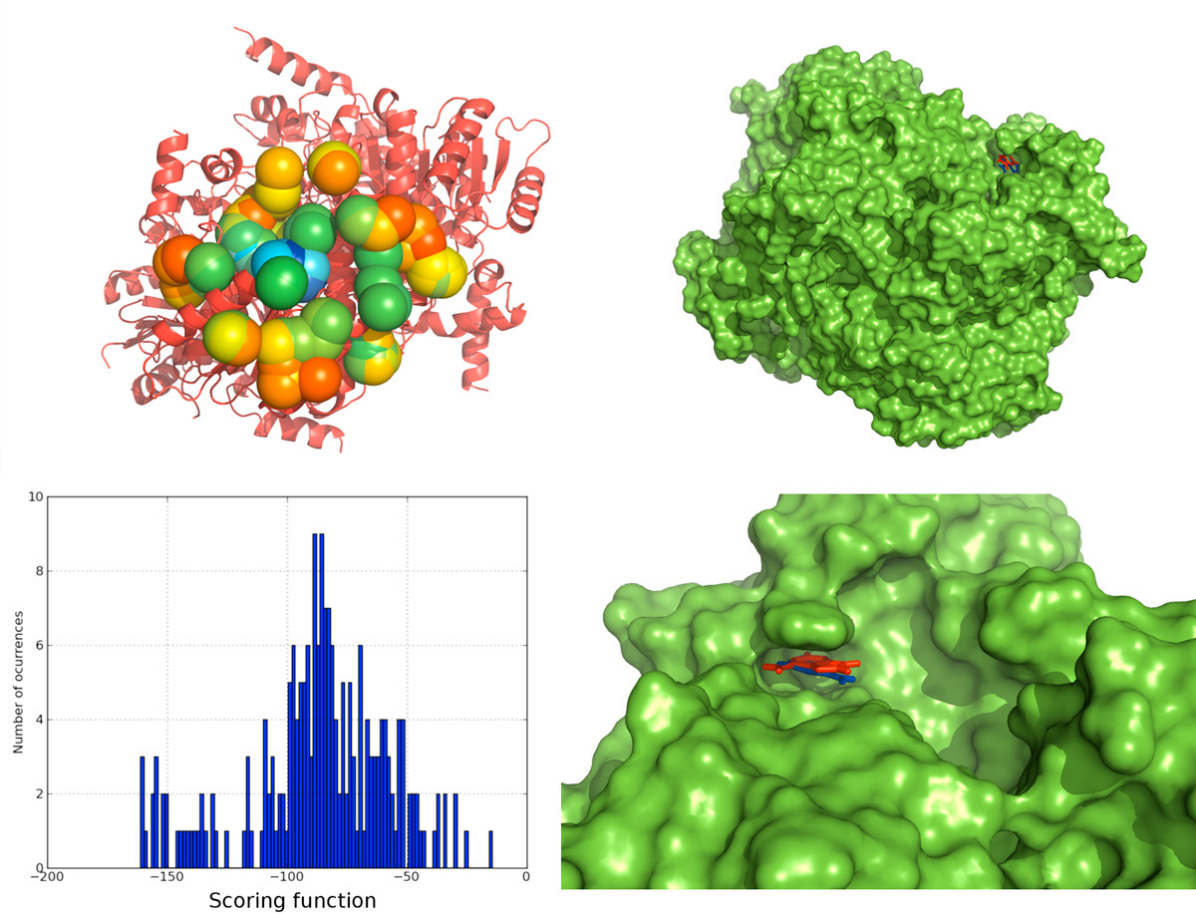
**Surface screening results for PDB:**1YXM. Surface screening results for PDB:1YXM. From up left to down right; a) beads represent protein spots and the color of each bead is related with the value of the scoring function, so colors from red to blue indicate lower values for the scoring function, b) representation of the PDB:1YXM protein surface, c) histogram with the distribution of scoring function values, d) red and blue molecules represent crystallographic and predicted pose for the ligand, RMSD is lower than 1 Angstrom.

**Figure 9:**
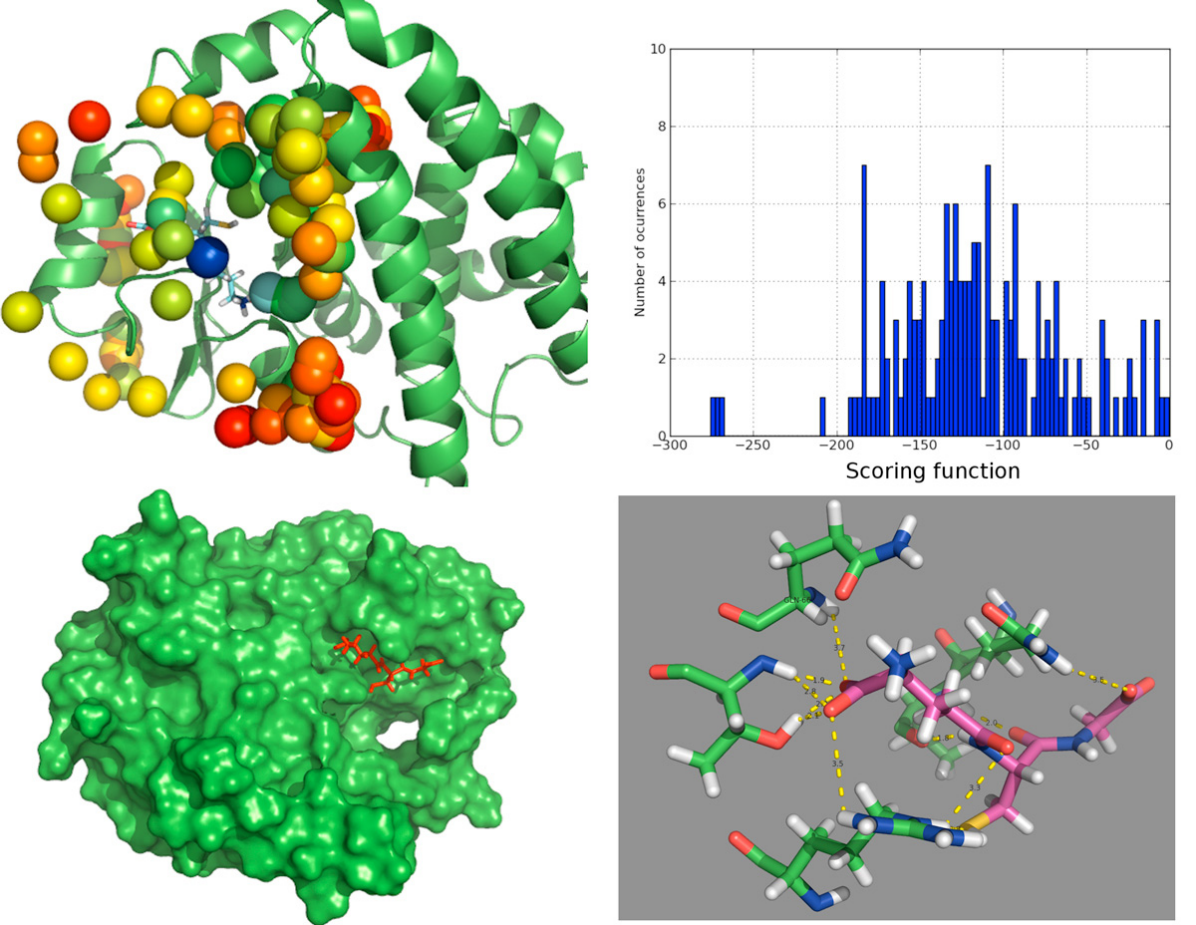
**Surface screening results for PDB:**1F3A. Surface screening results for PDB:1F3A. From up left to down right; a) beads represent protein spots and the color of each bead is related with the value of the scoring function, so colors from red to blue indicate lower values for the scoring function, b) histogram with the distribution of scoring function values, c) red molecule represents predicted pose for the ligand, RMSD is lower than 1 Angstrom, and d) depiction of the hydrogen bonds established by the ligand with the closest residues.

It has been reported in a previous study [32], cases where other computational approaches have tried to predict binding site location. We selected some of the cases where the previous methods [32] fail to provide accurate predictions and tested with *BINDSURF*. For peroxisomal trans 2-enoyl CoA reductase (PDB:1YXM), the binding site was reported in the mentioned study to be too small for predictions, but *BINDSURF *could find it efficiently, as shown in Figure 8. For Murine class alpha glutathione S-transferase A1-1 (PDB:1F3A), it was reported in the same study that the ligand would bind at the edge of the pocket but not inside the pocket. We performed the calculations with *BINDSURF*, as shown in Figure 9, and 9 could find both the binding site and binding pose efficiently.

**Figure 10:**
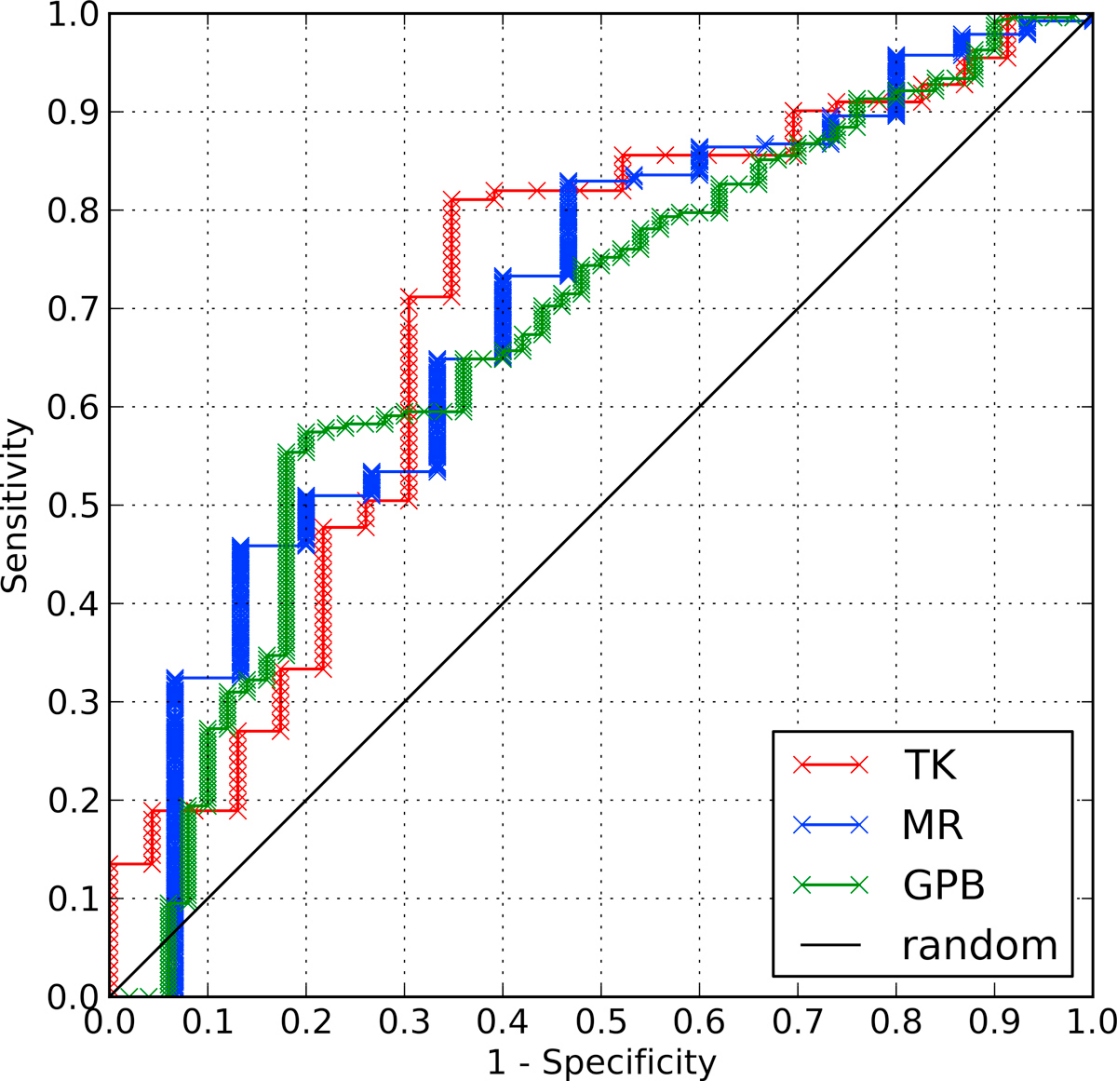
**ROC plots for DUD data sets**. ROC plots for the targets of the DUD data set TK (red), MR (blue) and GPB (green). Diagonal line indicate random performance. Obtained values for AUC are 0.700, 0.695 and 0.675, respectively.

Finally, we performed calculations to check the effectiveness of *BINDSURF *in the direct prediction of binding poses. For this purpose there are standard tests, like the Directory of Useful Decoys (DUD) [33], where VS methods check how efficient they are in differentiating ligands that are known to bind to a given target, from non-binders or decoys. Results for three different DUD datasets are shown in the ROC curves of Figure 10. Given the results obtained for the DUD datasets TK, MR and GPB, and characterized by the value of the area under the curve (AUC) for each ROC curve, it could be said that, on average, *BINDSURF *performs similarly than other docking methods reported for these datasets [34].

Nevertheless, it is clear that there is still room for improvement in the scoring function that *BINDSURF *uses, and in its energy optimization method (Monte Carlo), since both affect directly to the effectiveness of the direct prediction of binding poses.

## Conclusions

In this work we have presented the *BINDSURF *program. We have shown the details of its modular design, so other users can modify it to suit their needs.

In view of the results obtained, we conclude that *BINDSURF *is an efficient and fast methodology for the unbiased determination on GPUs of protein binding sites depending on the ligand. It can also be used for fast pre-screening of a large ligand database, and its results can guide posterior detailed application of other VS methods. Its utilization can help to improve drug discovery, drug design, repurposing and therefore aid considerably in clinical research.

In the next steps we want to substitute the Monte Carlo minimization algorithm for more efficient optimization alternatives, like the Ant Colony optimization method, which we have already implemented efficiently on GPU [35] and implement also full ligand and receptor flexibility.

Lastly, we are also working on improved scoring functions to include efficiently metals, aromatic interactions, and implicit solvation models.

The program code is available upon authors' request.

## List of abbreviations used

The list of abbreviations used is the following: GPU: (Graphics Processing Unit); VS: (Virtual Screening); ES: (Electrostatic); VDW: (Van der Waals); HBOND: (Hydrogen bonds).

## Competing interests

The authors declare that they have no competing interests.

## Authors' contributions

ISL designed and implemented the BINDSURF system described in this paper, performed the tests and calculations needed for the study and participated in the drafting of the manuscript. JMC contributed to the parallel design of the surface screening algorithm and helped to draft the manuscript technical part. HPS and JMG conceived of the study, participated in the design of the study and the algorithms, and in the coordination, drafting and final writing of the manuscript. All authors read and approved the final manuscript.
